# Performance Research of Natural Mica Modified with Zirconium-Based Metal–Organic Frameworks for an Epoxy Resin Anti-Corrosion Coating

**DOI:** 10.3390/molecules28207106

**Published:** 2023-10-16

**Authors:** Lijuan Zhu, Chun Feng, Bokai Peng, Xuezhi Hui, Xiaofeng Bai, Zongxue Yu

**Affiliations:** 1 Tubular Goods Research Institute, China National Petroleum Corporation, Xi’an 710077, China; 2 State Key Laboratory for Performance and Structure Safety of Petroleum Tubular Goods and Equipment Materials, Xi’an 710077, China; 3 Key Laboratory of Petroleum Tubular Goods and Equipment Quality Safety for State Market Regulation, Xi’an 710077, China; 4 School of Chemistry and Chemical Engineering, Southwest Petroleum University, Chengdu 610500, China; 5 Petrochina Changqing Oilfield Company, China National Petroleum Corporation, Xi’an 710021, China

**Keywords:** mica, polyethyleneimine, epoxy resin, metal–organic framework, anticorrosion coating

## Abstract

A new composite material made from mica and a metal–organic framework (MOF) has been developed to improve the anticorrosive capabilities of epoxy resin coatings. The layered mica was loaded with denser and more uniform UIO-66 nanoparticles after modifying the composite with polyethyleneimine (PEI). The composites were used as fillers to prepare epoxy coatings that exhibited long-lasting active (labyrinth effect produced by mica) and passive (pH-sensitive release of corrosion inhibitors) corrosion protection. Settling experiments showed that polyethyleneimine improved the composites’ compatibility in epoxy resin. After being immersed in a 3.5 wt.% NaCl solution for 60 days, the adhesion of PMC–UIO@MBT/EP increases to 9.01 MPa, while the water absorption rate only reaches 2.57%. It indicates that the coating has good barrier properties and stability. After being soaked in a 3.5 wt.% NaCl solution for 60 days at pH = 7, PMC–UIO@MBT/EP exhibits high low-frequency impedance (8.30 × 10^8^ Ω), as demonstrated by the electronic impedance spectrum (EIS). In addition, the coating also exhibited the highest low-frequency impedance after 30 days in 3.5 wt.% NaCl solution at pH = 11.

## 1. Introduction

In recent years, the excellent physical blocking performance of 2D layered materials in anticorrosive coatings has been widely used in epoxy resin coatings, such as graphene [1,2], oxide graphene [3,4], hydroxyapatite [5,6], boron nitride [7] and MXenes [8]. However, two-dimensional materials such as graphene and MXenes have difficulty in preparation and high costs, making them unsuitable for large-scale industrial applications. In addition, their good conductivity may cause the electrical corrosion of metal substrates, thereby accelerating metal corrosion and shortening the service life of the coating [9]. Especially for graphene, once the coating is damaged, the graphene may promote metal corrosion by inducing the galvanic potential change on the interface of the graphene/substrate [10]. There is much research on other two-dimensional layer-like materials in the anticorrosive coating. Boron-nitride (H-BN), also known as “white graphene”, has characteristics similar to graphenes [11], such as insulative [12] and mechanical properties [13] and thermal conductivity [14]. However, boron nitride is attracted by the layer of Van der Waals forces. It is not straightforward to make the boron nitride peel into several layers of nanosheets [15]. It has excellent barrier properties in the epoxy coating as a new two-dimensional transition metal carbide (transition metal carbide/nitride) [8]. However, MXenes peeling into nanosheets must be etched through HF (toxic chemicals). Therefore, it is essential to choose a two-dimensional material to delay corrosion and reduce the epoxy coating cost [16].

Mica is a piece of natural layers like clay. Its chemical formula is KAl_2_ (Si_3_AlO_10_ (OH)_2_) and is not sandwiched by an octagonal aluminum (Al_2_O_3_) layer on two identical tetragonal silicon oxide layers between other metal oxides. Natural mica exists widely in nature with low prices. Mica demonstrates some exceptional performance compared to other 2D layered materials, such as high transparency [17], ultraviolet shielding [18], electrical insulation [19], temperature stability [20,21], chemical stability [21], and biocompatibility [19]. These superior performances have made mica widely studied in some fields. In order to exploit the excellent properties of mica, it is crucial to exfoliate natural mica into nanosheets with fewer layers. Common methods for exfoliation of natural mica include tape adhesion [22], solvent heat [23], and ion intercalation (metal ion intercalation [11] and cetyltrimethylammonium bromide (CTAB) intercalation [18]). Due to the excellent physical barrier performance of two-dimensional layer materials, they are widely used in anticorrosive coating. After peeling, the single-layer or small-layer mica nanosheets also have excellent blocking performances. In addition, mica has electrical insulation, so mica has outstanding advantages in anticorrosive coating; it is impossible to perform electrochemical corrosion with a metal base. Therefore, in recent years, mica has had more research in anticorrosive coating. Ding et al. [11,24] used Li^+^ inserting mica, peeling the mica to the 1–5 layer under hydrothermal reaction. As a result, the corrosion rate of the cloud-based epoxy resin coating decreased by 30 times compared to pure epoxy resin coating. In addition, they also prepared a bionic epidermal appearance of smart mica steel surface anti-corrosion coatings. The results showed that the protection efficiency of the coating reached 99.8%, which was much higher than most of the promising graphene-based anticorrosive coatings. Meng et al. [25] prepared epoxy resin-based modified mica coatings and the degradation of the protective properties of the coatings and the failure of the coatings under the action of marine alternating hydrostatic pressure (AHP) were slowed down. Moreover, they concluded that the chemical bonding at the interface of mica and epoxy resin improved the dispersion of mica particles, reduced the formation of initial surface defects, and improved the denseness and mechanical properties of the coating. Li et al. [26] used SiO_2_, graphene oxide (GO), and lamellar mica powder (MP) to create a long-lasting superhydrophobic coating that remains hydrophobic in harsh chemical environments. The process is simple and inexpensive. This research result provides an idea for developing mica-based materials for corrosion-resistant coatings. In a recent study, Bai et al. [27] anchored zinc molybdate to a mica surface. The zinc molybdate acted as an active inhibitor in the coating and the coating showed good corrosion resistance. Similar studies are shown in Table 1.

MOFs are a new class of nanoporous structures consisting of metal ions as precursors and organic compounds as ligands. MOFs have properties such as high surface area, ionic conductivity, and high durability in harsh chemical environments [28,29], which equip them with an excellent potential for development in the fields of gas storage [30], drug delivery [31], and catalysis [32]. In recent years, it has been applied to anti-corrosion coatings. Al Kiey et al. [33] successfully prepared three metal–organic frameworks (MOFs), Fe-BTC, Mn-BTC, and Co-BTC, as corrosion inhibitors. Their study proved that Mn-MOF has the best retarding ability. Ren et al. [34] prepared an MOFs-based anticorrosive material containing the corrosion inhibitor ZnG@ZIF-8 by using ZIF-8 as a matrix for encapsulating the corrosion inhibitor. Their study showed that adding ZnG@ZIF-8 composite could effectively enhance the coating’s corrosion protection and mechanical properties. Lashgari et al. [35] synthesized composites of ZIF-67 and GO and applied them with epoxy coatings. After corrosion, cobalt ions released from the GO/ZIF-67 composite could form a protective film based on Co(OH)_2_ on the cathode, which conferred self-healing properties to the coating. Chen et al. [36] prepared NH_2_-MIL-125(Ti) with boron nitride composites in epoxy resin, which also showed excellent corrosion protection. It is mainly due to NH_2_-MIL-125(Ti) as a corrosion inhibitor for nanocontainer-loaded MBT. Therefore, applying MOFs in coatings is essential and it is especially critical to enhance the loading of MOFs on 2D materials. In this study, polyethyleneimine modification of UIO-66 significantly enhanced the loading capacity of UIO-66 on mica. This facilitates the enhancement of the dispersion of the composite in the epoxy resin and the loading of corrosion inhibitors compared to other studies on MOFs-based anti-corrosion coatings.

**Table 1:** A comparison table of various studies on anti-corrosion coatings made with mica.

Study	Findings
Ding et al. [24]	A coating resembling skin structure was made to improve barrier properties.
Yu et al. [37]	Synthesis of oriented polyaniline nanofibres doped with phytic acid on a mica surface for ultra-high corrosion protection of epoxy coatings. In addition, the synergistic effect of polyaniline nanofibres and mica enhanced the adhesion of the coating.
Bai et al. [27]	Polyethyleneimine as binder and zinc molybdate as additive sericite were modified. This composite gives the epoxy coating self-healing properties.
Xiao et al. [38]	MNs surfaces can be made superhydrophobic by interacting them with natural laccase phenol (Ur) catechol groups.
Ye et al. [39]	Tannic acid modified mica can enhance the dispersibility of mica in epoxy resin.

Our present study uses a modified method of ultrasonically exfoliating natural mica to obtain a monolayer or layer of fewer mica nanosheets. 2-mercaptobenzothiazole (MBT) is loaded into a UIO-66 nanocontainer with mica nanosheets to act as a corrosion inhibitor. The corrosion inhibitor’s release is achieved by disrupting the crystal structure of UIO-66 in a corrosive environment. The combination of the excellent barrier properties of mica and the action of corrosion inhibitors increases the corrosion resistance of the coating over time. Polyethyleneimine (PEI) is added to improve the dispersion of the composite in the epoxy resin and provide pitting resistance to the composite coating, thereby enhancing its corrosion resistance. The resulting nanocomposite is named PMC–UIO@MBT. The PMC–UIO@MBT nanocomposite was used as a filler in the epoxy resin coating. In summary, it modified mica by introducing UIO-66 containing amino groups and polyethyleneimine as fillers. It enhances the dispersibility of the mica in the epoxy resin. At the same time, UIO-66 can be used as a nano-vessel loading corrosion inhibitor to give the epoxy coating a self-healing effect. PMC–UIO@MBT nanocomposite should hold great promise in synergistically achieving long-term corrosion resistance of epoxy coatings.

## 2. Results and Discussion

### 2.1. The Characterization of PEI-MC/UIO@MBT Nanocomposite 

#### 2.1.1. XRD Analysis

**Figure 1:**
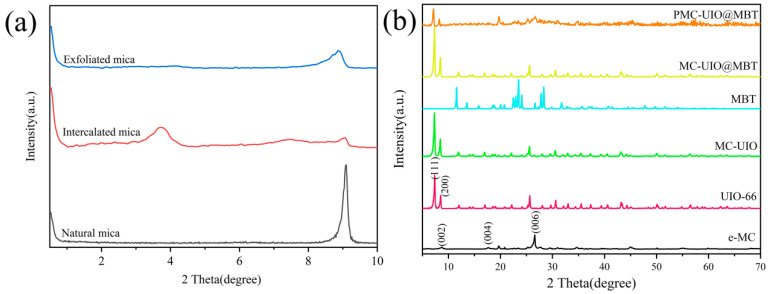
(**a**) Small-angle XRD patterns of natural mica, mica after CTAB intercalation, and mica after being exfoliated, respectively; (**b**) large-angle XRD patterns of e-MC, UIO-66, MBT, MC–UIO, MC–UIO@MBT, and PMC–UIO@MBT.

The experimental results of XRD are shown in Figure 1. Small angle XRD (2θ = 0.5–10) was performed to confirm that the natural mica was exfoliated into a single-layer nanosheet or one with a few layers, as shown in Figure 1a. The dense negative charge on the surface of the mica and the attractive forces formed by the cations between the mica layers are responsible for the difficulty in exfoliating the mica. In the small-angle XRD results of natural mica with a sharp peak at 2θ = 9.1°, the intensity of the exfoliated mica peak diminishes significantly, indicating a reduction in thickness after exfoliation [18]. In addition, CTAB is embedded in intercalated layers of natural mica to widen the mica layer spacing. After the intercalation, a new diffraction peak appears in the small angle XRD of the mica at approximately 2θ = 3.6° in Figure 1a. After the Bragg formula, the mica layer spacing was expanded to approximately 2.41 nm. The expansion of the mica layer spacing facilitated ultrasonic exfoliation into nanosheets with a few layers. In the XRD patterns (5–70°), 2θ = 8.7°, 2θ = 17.8°, and 2θ = 26.6° correspond to the (002), (004), and (006) crystal planes of mica, respectively. In addition, no new diffraction peaks were generated in the mica obtained after ultrasonic stripping. The above results confirm that the stripping of natural mica and ultrasonic stripping does not affect the crystal structure of mica.

In Figure 1b, the diffraction peaks of UIO-66 at 2θ = 7.4° and 2θ = 8.5° correspond to the (111) and (200) crystal planes, respectively. This confirms the successful synthesis of UIO-66, consistent with previous research cited in the references [40,41,42]. The similarity to the diffraction peaks of UIO-66 is evident in the plots of MC–UIO and MC–UIO@MBT, indicating that the mica surface is loaded with UIO-66 crystals. The diffraction peak of MBT disappears in MC–UIO@MBT due to the successful encapsulation of MBT corrosion inhibitor in UIO-66. The XRD pattern of PEI-modified composites does not show any significant peaks due to the amorphous nature of PEI, a phenomenon reported in the literature before [43]. Therefore, the PEI modification does not change the crystal structure of the composites. 2θ = 8.7° shows the disappearance (002) of the mica and part of the characteristic peak of UIO-66, which is due to the amorphous nature of PEI and proves, from another point of view, the success of the PEI modification of the composites. Furthermore, the appearance of a new diffraction peak at 19.7° implies the recovery of the mica structure.

#### 2.1.2. FTIR Analysis

**Figure 2:**
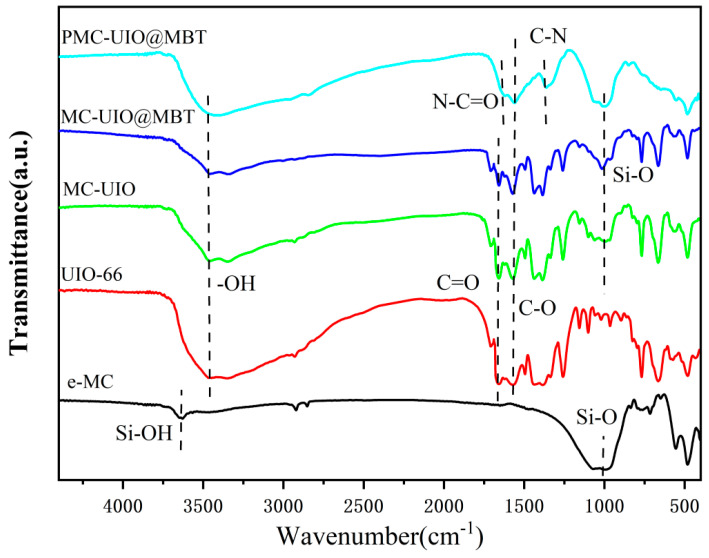
FTIR patterns of e-MC, UIO-66, MC–UIO, MC–UIO@MBT, and PMC–UIO@MBT.

Figure 2 shows the FTIR spectra of the various synthetic materials. In the FTIR spectrum of mica, the peak at 3629 cm^−1^ is attributed to the Si-OH stretching vibration of the mica. The peaks at approximately 1004 cm^−1^ and 470 cm^−1^ correspond to the stretching vibrations of the Si–O–Si group [44,45,46]. The use of CTAB to intercalate the mica and ultrasonically exfoliate it did not change the chemical groups of the mica. For UIO-66, the spectrum of the zirconium oxygen cluster consists of peaks from 600 cm^−1^ to 800 cm^−1^ that relate to the longitudinal and transverse modes of Zr-O. In particular, the spectrum of UIO-66 at 1380 cm^−1^ and 1580 cm^−1^ includes symmetric and asymmetric stretching vibrational peaks of the carboxyl group due to the stretching of the C–C aromatic ring. This confirms the presence of 2-amino terephthalic acid as an organic ligand in the structure of UiO-66. The peak at 1669 cm^−1^ is also associated with stretching vibrations of the C=O group, suggesting the bonding of the Zr metal through coordination with the organic ligand [47]. The infrared spectra of MC–UIO and MC–UIO@MBT are similar in shape to UIO-66. The absence of Si-OH characteristic peaks in the FTIR spectra of MC–UIO, MC–UIO@MBT, and PMC–UIO@MBT may be attributed to the amidation reaction between the hydroxyl group of mica and the amino group of UIO-66 [12,48]. However, the characteristic peak of the hydroxyl group at 3469 cm^−1^ is significantly weaker for MC–UIO and MC–UIO@MBT. In addition, the characteristic peak of the Si–O–Si mica group appears near 1004 cm^−1^. The results show successful loading of UIO-66 on two-dimensional layered mica. The introduction of PEI, the formation of a new C=O by reaction with UIO-66 appears at 1639 cm^−1^, and the peak at 1371 cm^−1^ are due to the C–N stretching vibration [49]. In addition, the characteristic peaks of mica were retained in the FTIR spectrum of PMC–UIO@MBT. The above-discussed results can prove the successful synthesis of PMC–UIO@MBT, which corresponds to the experimental results of XRD, SEM, and TGA.

#### 2.1.3. TGA Analysis

**Figure 3:**
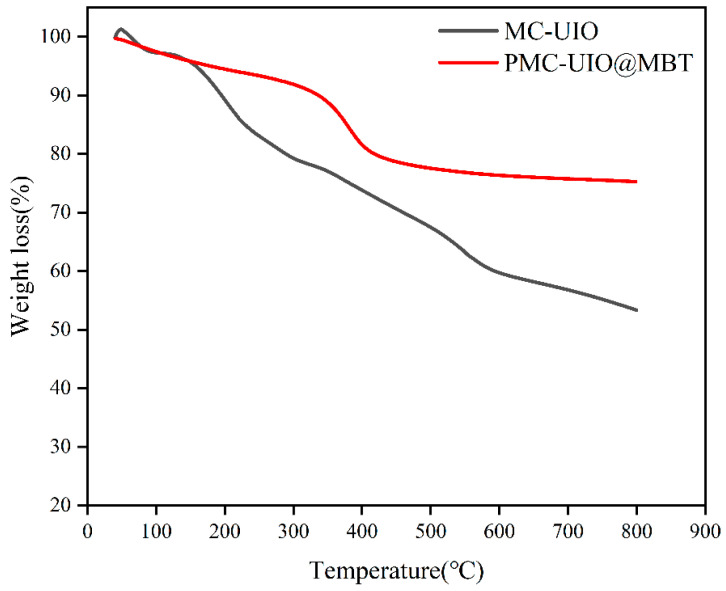
TGA results for PMC–UIO@MBT, MC–UIO material.

The TGA curves for PMC–UIO@MBT and MC–UIO are shown in Figure 3. As can be seen in the TGA results, both PMC–UIO@MBT and MC–UIO have less than 50% mass loss after heating to 800 °C, which is attributed to the high-temperature resistance of the mica. Due to water evaporation, there is no significant difference in their thermal decomposition curves below 150 °C. After 150 °C, the thermal decomposition curves of PMC–UIO@MBT can be divided into the following three stages. Firstly, between 200 °C and 300 °C, this weight loss is due to the decomposition of the corrosion inhibitor MBT. The composite has successfully incorporated the corrosion inhibitor MBT. Then, between 300 °C and 400 °C, the PMC–UIO@MBT composite exhibited a more significant weight loss with a weight change of 12.97%; It denotes the thermal decomposition of PEI molecules [50]. Finally, the weight loss between 450–600 °C was caused by the decomposition/collapse of the UIO-66 metal–organic framework [51]. The weight loss in the three stages confirms the successful preparation of PMC–UIO@MBT composites. In conclusion, the results of the TGA can indicate the successful preparation of the PMC–UIO@MBT composite.

#### 2.1.4. BET Analysis

**Figure 4:**
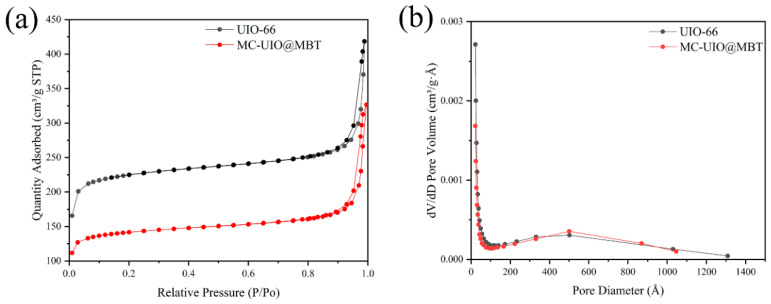
(**a**) Nitrogen adsorption/desorption isotherms for UIO-66 and MC–UIO@MBT; (**b**) Pore size distribution of UIO-66 and MC–UIO@MBT.

The N_2_ adsorption–desorption isotherms of the samples and the pore size distribution are shown in Figure 4. In the N_2_ adsorption–desorption isotherms, UIO-66 and MC–UIO@MBT are typical type IV reversible isotherms showing H3-type hysteresis loops. This indicates that the pore structure is not regular on both composites and that the adsorption has not reached saturation. Table 2 shows the property parameters of the composites. Due to the high specific surface area of UIO-66, it is feasible to load the corrosion inhibitor as a nanocontainer. The BET surface area and pore volume of UIO-66 are higher than MC–UIO@MBT, indicating successful encapsulation of the corrosion inhibitor.

#### 2.1.5. Corrosion Inhibitor Release Performance Testing

**Figure 5:**
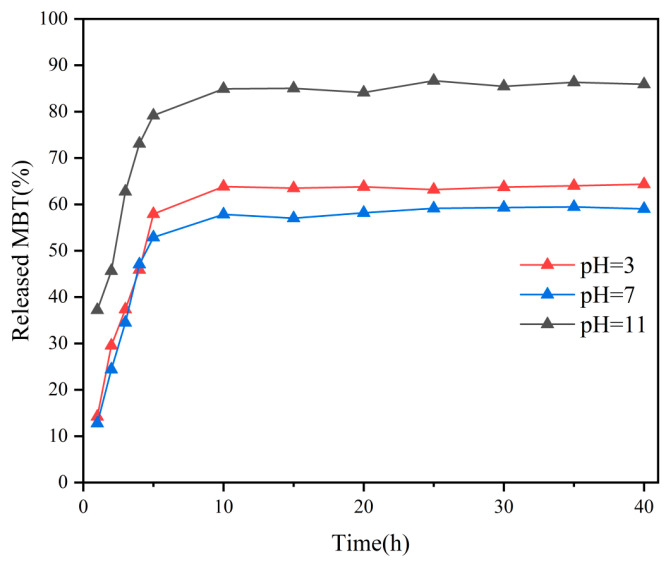
Profiles of MBT release from UIO-66 at different pH values measured with UV–VIS spectroscopy.

In the structure of the UiO-66 nanoparticles, each Zr_6_O_4_(OH)_4_ metal center is linked to an adjacent metal center by 12 organic ligands. The UiO-66 nanoparticles decompose in an aqueous solution by ligand substitution and hydrolysis reactions. In the presence of NaOH and HCl solutions, ligand displacement and hydrolysis reactions lead to structural defects in UiO-66, resulting in the easier release of the compound. To investigate the release kinetics of MBT at different pH conditions, UV–VIS tests of PMC–UIO@MBT composite materials at 3.5 wt.% NaCl (pH = 3, pH = 7 and pH = 11) were conducted. Figure S1 shows the UV–VIS absorbance curve of the corrosion inhibitor MBT. The wave peak appears around 320 nm, the UV–VIS absorption peak of MBT, consistent with the previous research [52]. As shown in Figure 5, the absorbance of all samples showed a trend of increasing and then stabilizing under different conditions (pH =3, pH =7, and pH =11) with increasing experimental time. The difference is that at pH = 11, the MBT was released rapidly and in large quantities. After 40 h, the MBT release rates for the three pH conditions were 64%, 59%, and 86%, respectively. This indicates that the PMC–UIO@MBT composite is pH-responsive and enables the rapid and significant release of corrosion inhibitors under alkaline conditions. 

**Figure 6:**
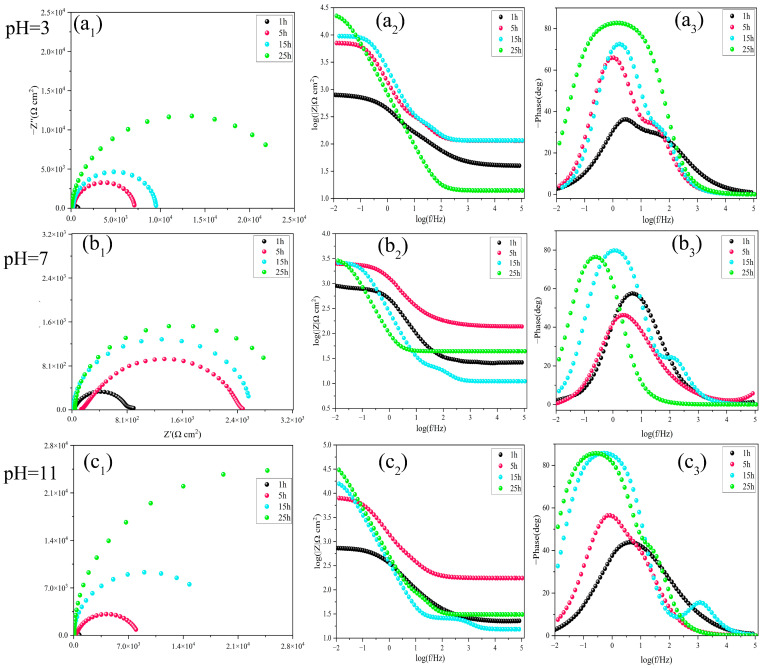
Bode and Nyquist plots of carbon steel after different immersion times in 3.5 wt.% NaCl solution containing PMC–UIO@MBT composite at (**a_1_**–**a_3_**) pH = 3, (**b_1_**–**b_3_**) pH = 7, and (**c_1_**–**c_3_**) pH = 11.

Bare carbon steel electrodes were immersed in a PMC–UIO@MBT solution of 3.5 wt.% NaCl at different pH conditions. The electrochemical impedance spectroscopy (EIS) test revealed the suppression of MBT corrosion inhibitor release on the surface of the metal substrate. The results are illustrated in Figure 6. In acidic and neutral environments, the low-frequency impedance modulus(|Z|) increases with soaking time and the Nyquist plot of the impedance arc radius tends to increase with longer soaking times. However, this change was not significant in the later stages of immersion. Under alkaline conditions, the low-frequency impedance modulus(|Z|) increases with increasing immersion time. In addition, the magnitude of the low-frequency impedance modulus after 25 h immersion under acidic and alkaline conditions is 2.24 × 10^4^ Ω cm^2^ and 3.04 × 10^4^ Ω cm^2^, respectively. This suggests that composite materials can more effectively prevent metal corrosion in both acidic and alkaline environments.

#### 2.1.6. Dispersion Stability Testing of Composite Materials

**Figure 7:**
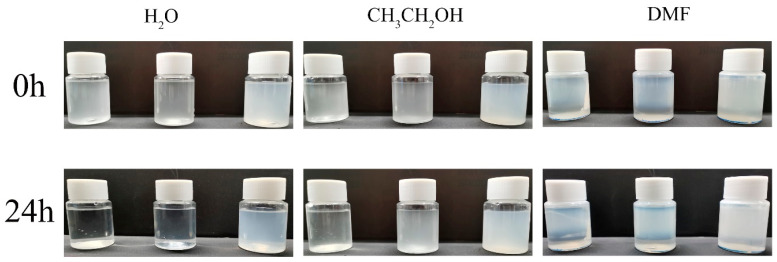
Photos of NMC, e-MC, and PMC–UIO@MBT materials in H_2_O, CH_3_CH_2_OH, and DMF solutions for 24 h, respectively.

**Figure 8:**
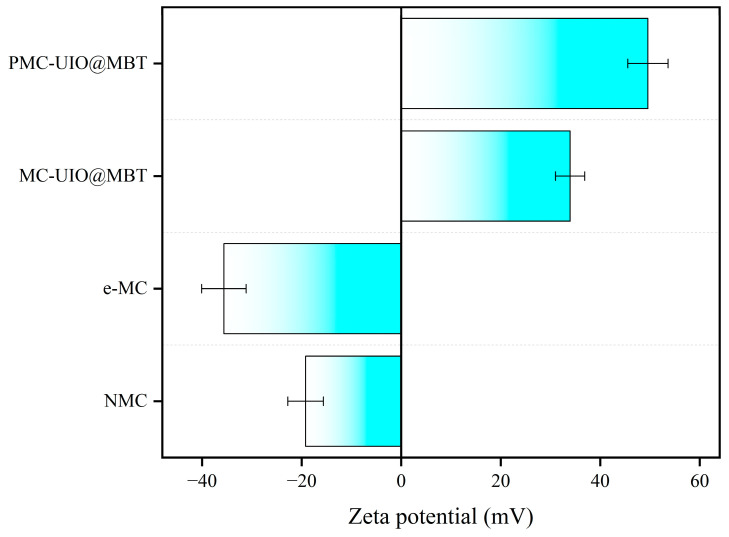
Zeta potential values for NMC, e-MC, MC–UIO@MBT, and PMC–UIO@MBT composites.

Figure 7 illustrates the dispersion stability of the material in the solvent. Three solvents, H_2_O, CH_3_CH_2_OH, and DMF, were used to dissolve the composites (from left to right in the figure: NMC, e-MC, and PMC–UIO@MBT) and the settling of the composites after 24 h of standing was observed to obtain the dispersion stability of the materials. The dispersion stability of all three materials in the different solvents varied. The natural mica (NMC) settled ultimately in all solvents after 24 h of standing and the supernatant was clear. After ultrasonic stripping, the mica (e-MC) showed relatively good dispersion stability. Although precipitation occurred in both water and DMF, no ethanol precipitation occurred. The dispersion of mica was improved after it was exfoliated into nanosheets. Interestingly, the dispersion of the PMC–UIO@MBT composites in water and DMF was improved after modification of the composites by PEI. This may be due to the similar solubility of the modified polyethyleneimine and UIO-66 amino groups with the solvent. The Zeta potential measurement helps determine the level of dispersion stability of a material [53]. The above conclusions can likewise be obtained from Figure 8. In addition, the zeta potential changes from harmful to positive values after the composite is modified by polyethyleneimine, a change that has also been reported in the literature [18]. The surface of the mica has a negative charge [54]. After adding UIO-66, the composite material has a site to absorb hydrogen ions and becomes positively charged. The PMC–UIO@MBT composite surface is positively charged because of PEI’s H+ doping. The absolute values of PMC–UIO@MBT composites also increase with the increase in absorbed H+ sites. This also indicates that PEI’s surface modification of the composites was successful.

#### 2.1.7. Micromorphology of Composite Materials

**Figure 9:**
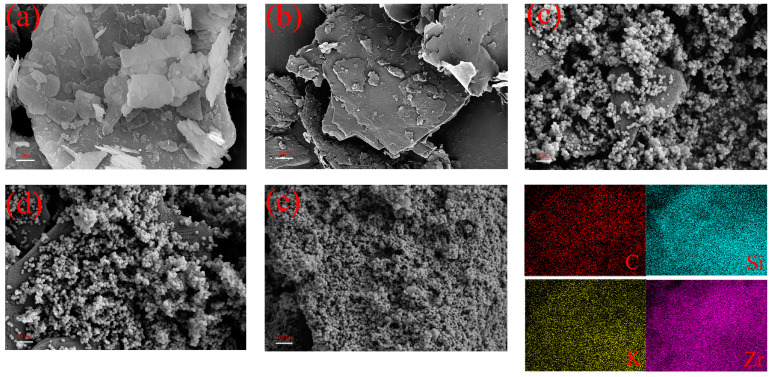
SEM images of (**a**) NMC, (**b**) e-MC, (**c**) MC–UIO, (**d**) MC–UIO @MBT, and (**e**) PMC–UIO @MBT.

The microscopic surface morphology of the composites was analyzed using SEM. As shown in Figure 9a, the natural mica exhibits a flake morphology with neat and smooth edges and no apparent adhesions on the flakes. The natural mica flakes are heavily agglomerated. Figure 9b shows the exfoliated mica flakes with curled and fluffy edges and a significant reduction in agglomerate thickness. e-MC is also typical of the flake morphology. In addition, the lateral dimensions of e-MC are reduced compared to the lateral dimensions of NMC. Figure 9b shows the microscopic morphology of MC–UIO loaded with UIO-66 particles on a laminar structure of mica. In addition, the EDS pattern shows homogeneous dispersion of element Zr on the composite, indirectly proving the heavy loading of UIO-66 on the lamellar material. In addition, the EDS pattern shows homogeneous dispersion of element Zr on the composite, indirectly proving the heavy loading of UIO-66 on the lamellar material. UIO-66 shows a uniform size and regular polyhedral shape on the lamellar structure. As shown in Figure 9d, for MC–UIO@MBT, UIO-66, as a nanocontainer loaded with MBT corrosion inhibitor, also did not change its microscopic morphology. Interestingly, a more dense and homogeneous UIO-66 was loaded on the polyethyleneimine-modified composite, as shown in Figure 9e. According to the EDS diagram, the phenomenon aligns with previous studies [55,56]. The loading of corrosion inhibitors in the composite is facilitated by using UIO-66 as a nanocontainer.

### 2.2. Performance Characterisation of PMC–UIO@MBT/EP Composite Coatings

#### 2.2.1. Micromorphology of Coated Sections

**Figure 10:**
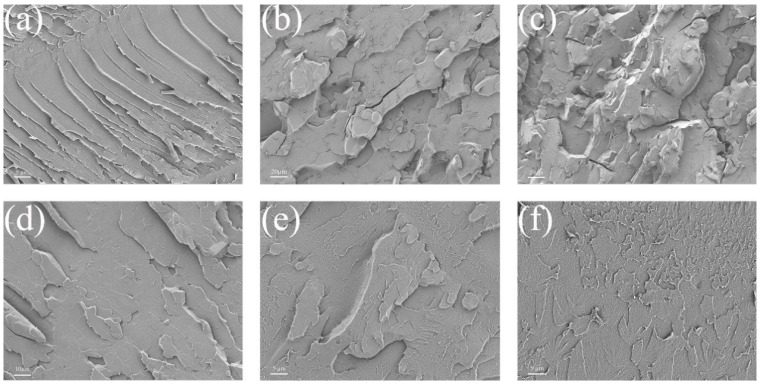
Fracture surfaces of (**a**) pure EP, (**b**) NMC/EP, (**c**) e-MC/EP, (**d**) MC–UIO/EP, (**e**) MC–UIO@MBT/EP, and (**f**) PMC–UIO@MBT/EP.

All the composite coatings’ fractures were compared using scanning electron microscopy to investigate the compatibility of PMC–UIO@MBT/EP composite coatings with epoxy resin coatings. Figure 10 shows the results. The fractures of pure EP have a smooth surface, a neat crack direction, and a stepped-shaped structure, as shown in Figure 10a. This is due to the high brittleness of the pure epoxy resin. After the addition of mica, the fracture surface of the coating becomes significantly rougher and the direction of the cracks is not oriented. As shown in Figure 10b,c, the fracture surface has microscopic holes and tear lines, which is a result of the organic solvent evaporating during the high-temperature curing of the coating, leading to defects in the coating [57]. In addition, the agglomeration of mica in the resin may also contribute to this phenomenon. This is detrimental to the barrier of the coating to corrosive media. However, in the cross-sectional view of MC–UIO/EP (Figure 10d), the fracture is relatively flat and homogeneous compared to Figure 10b,c, indicating that the introduction of UIO-66 has improved the compatibility of the mica in the coating. In addition, the addition of MBT corrosion inhibitor did not alter the fracture morphology of the coating and the fractures of MC–UIO@MBT/EP (Figure 10e) were similar to those of MC–UIO/EP (Figure 10d). The fracture surface of PMC–UIO@MBT/EP (Figure 10f) appeared smooth, with no visible holes and no directional extension of cracks. This indicates that the amino groups introduced by UIO-66 and PEI together improve the compatibility of the mica in the resin, increasing the crosslink density and fracture toughness of the coating in favor of the barrier to the penetration of corrosive media.

#### 2.2.2. Compatibility of Composites in Epoxy Resins

**Figure 11:**
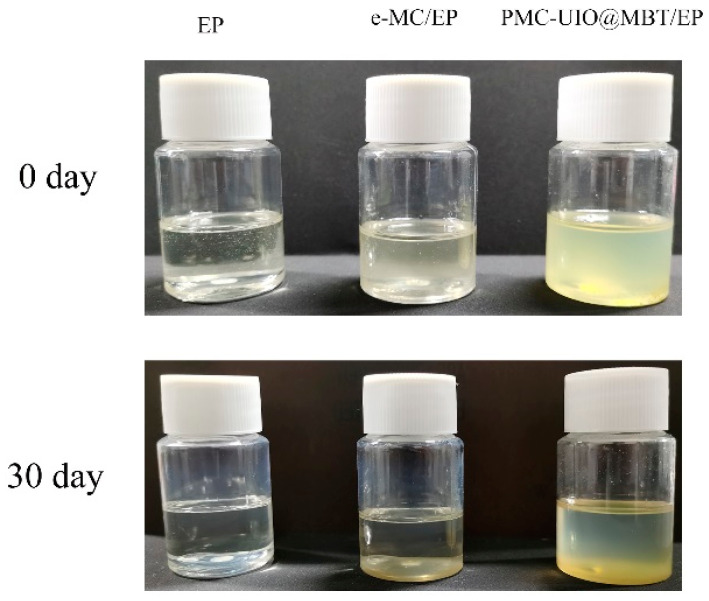
e-MC/EP and PMC–UIO@MBT/EP materials dissolved in epoxy emulsions settling test 0 and 30 days.

After dissolving the composites in the epoxy resin, the composites were observed to settle in the resting state. The compatibility of the composites in the epoxy emulsion was assessed based on the settling of the material. After 30 days of settling experiments, a photograph of the settling is shown in Figure 11. e-MC and PMC–UIO@MBT appear milky white and pale yellow when first dissolved in epoxy emulsions. The pure epoxy emulsions did not change color after 30 days. The epoxy emulsion with dissolved and e-MC disappears milky white after 30 days of settling and the epoxy emulsion becomes transparent. The e-MC had all settled to the bottom by the second day of standing. This indicates that the compatibility of e-MC in epoxy resins needs to be improved. After 30 days of settling, the epoxy emulsion with dissolved PMC–UIO@MBT was still cloudy and pale yellow with a small amount of sediment at the bottom of the bottle. Although the transparency of the epoxy emulsions increases, the difference is still significant compared to pure epoxy emulsions. This is attributed to the high reactivity of the amino group in UIO-66 and PEI with the epoxy group in the epoxy emulsion, resulting in excellent compatibility properties. The excellent compatibility of PMC–UIO@MBT in epoxy emulsions facilitates better barrier properties of the mica in the coating and enhances the long-term corrosion resistance of the coating.

#### 2.2.3. Adhesion Testing of Coatings

**Figure 12:**
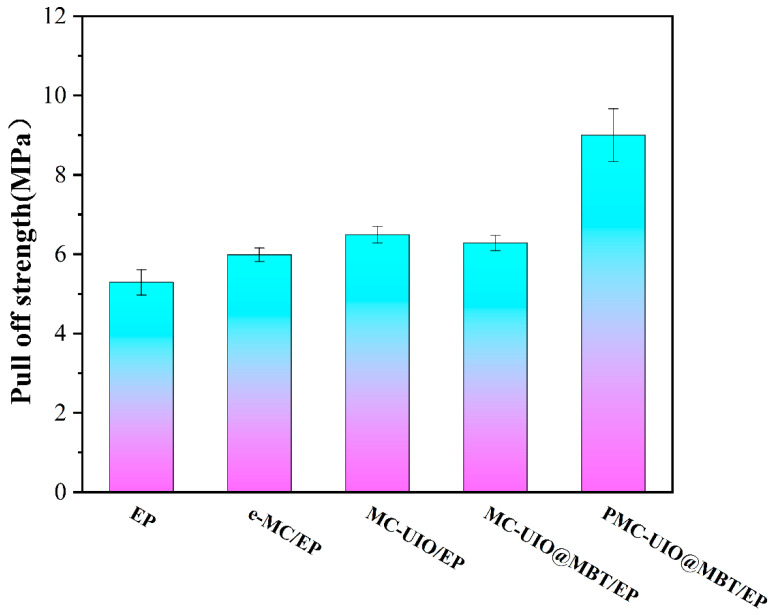
Adhesion values for composite coatings.

The strength of the bond between the coating and the metal matrix is an essential parameter in evaluating the stability of the coating. Stronger adhesion of the coating facilitates long-term corrosion resistance during the service life of the coating. The bond strength of the coating to the metal substrate using the mechanical pull-off coating test method and the results are shown in Figure 12. The pure epoxy adhesion of 5.29 MPa and the rapid failure of the pure epoxy in electrochemical impedance spectroscopy indicate that the bonding strength of the pure epoxy to the substrate is insufficient to support long-term corrosion resistance. Adding mica to the coating increases the adhesion to 5.98 MPa, which can be attributed to the Si-OH on the mica surface bonding with the Fe-O-Fe on the metal, forming a new Si-O-Fe [37]. The addition of UIO-66 improves the dispersion in the coating due to the introduction of amino groups, which increases the adhesion of MC–UIO/EP to 6.49 MPa. Since MBT is a plasticizer, it weakens the subvalent bonds between resin molecules, increases the mobility of resin molecular bonds, and reduces the crystallinity of resin molecules. Some of the MBT remaining outside the pores of UIO-66 enters the epoxy polymer’s molecular structure, reduces the resin’s rigidity, and causes some loss of mechanical properties (adhesion) of the coating [58,59]. In addition, the modification of PEI increases the dispersion of PMC–UIO@MBT in the resin again and introduces more -NH_2_ groups covalently bonded to the -OH on the mica. -NH_2_ reacts with the epoxy group, leading to interfacial interactions [60]. Consequently, PMC–UIO@MBT/EP exhibited a maximum adhesion force of 9.01 MPa through the combination of e-MC, UIO-66, and PEI. The surface morphology of all samples after pull-off experiments is shown in Figure S5.

### 2.3. Corrosion Resistance of PMC–UIO@MBT/EP Composite Coating

**Figure 13:**
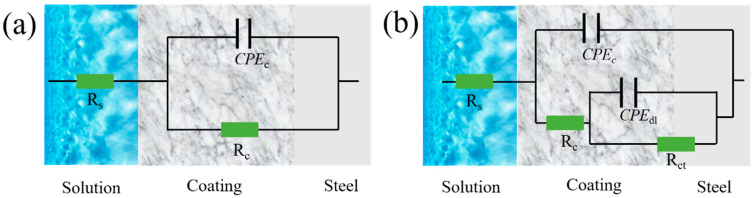
The equivalent electrical circuit for coatings in different stages. The one-time constant (**a**) and two times constant EEC models in the parallel (**b**) used for impedance results simulation.

The corrosion resistance of the coatings was tested using electrochemical impedance spectroscopy (EIS) in a conventional three-electrode mode with a counter electrode (platinum electrode), a reference electrode (KCl electrode), and a working electrode. The parameters were fitted and analyzed using Zsimpwin software for the EIS results. The EEC model of the fitted circuit is shown in Figure 13, which has the CDC codes R(QR) and R(Q(R(QR))). The fitted data are shown in Table S1. The goodness of fit χ^2^ is less than 10^−3^ for all the fitted data, which can prove the correctness of the fitted circuit.

Due to the undesirable behavior at the coating interface and the non-uniformity of the electrode sample surface, the heterogeneity index exponent (n) and the constant phase element (CPE) obtained from the conductance (Q) jointly represent the pure capacitance (C), with the relevant equation as follows [61]:

(1) $$Z_{\mathrm{CPE}}=\frac{1}{Q}{\left(jw\right)}^{-n}$$

where the value of the heterogeneity index exponent (n) is generally less than 1 and the value of Q corresponds to some value of n. Such a constant phase element (CPE) substitution gives a good match between the experimental data and the fitted data. The CPE_C_ and CPE_dl_ parameters obtained after constant phase element (CPE) substitution are analyzed separately to obtain the corrosion resistance mechanism of the coating.

In EIS testing, Nyquist and Bode’s diagrams are often used to visually compare the corrosion resistance of different coatings. Generally speaking, the larger the semicircle diameter of the Nyquist diagram, the more corrosion-resistant the coating is [62]. The low-frequency impedance modulus (|Z| = 0.01 Hz) can be used as an important indicator to evaluate the corrosion resistance of a coating in a Bode impedance diagram. The higher the low-frequency impedance modulus (|Z| = 0.01 Hz), the better the corrosion resistance [63]. In the Bode phase angle diagram, the higher the high-frequency phase angle (close to −90°), the better the integrity of the coating [64]. In addition, the breakpoint frequency (f_b_) is the frequency at a phase angle of −45° and is used to qualitatively compare the local exposure area of coatings on metal surfaces. This indicator is used to quickly evaluate the extent of damage to the coating. The higher the breakpoint frequency, the more severe the damage.

#### 2.3.1. Corrosion Resistance of the Coating in 3.5 wt.% NaCl Solution

**Figure 14:**
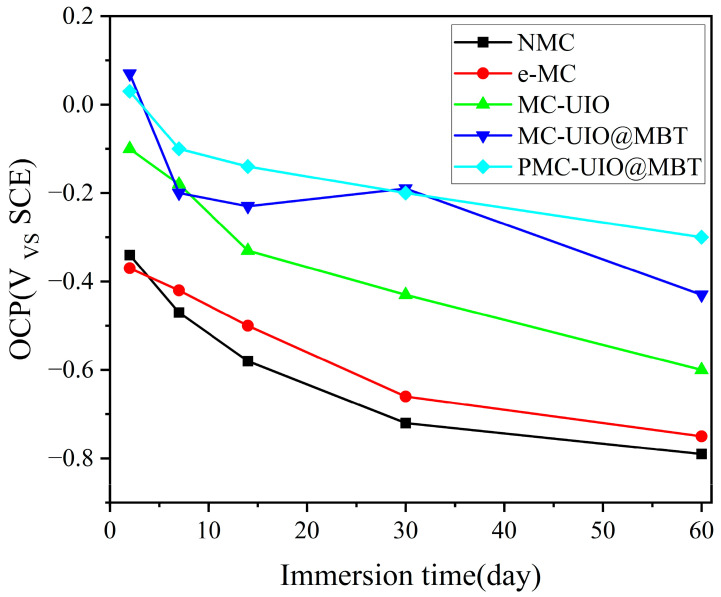
Trends in the open circuit potential for NMC/EP, e-MC/EP, MC–UIO/EP, MC–UIO@MBT/EP, and PMC–UIO@MBT/EP.

The long-term corrosion resistance of the composite coating was evaluated using open circuit potential (OCP). The coatings were immersed in a solution of 3.5 wt.% NaCl for 60 days and the open circuit potential was tested at different stages of immersion, as shown in Figure 14. The open circuit potentials of all the composite coatings showed a decreasing trend with increasing immersion time. The open circuit potential of the mica becomes larger after ultrasonic stripping relative to natural mica, which indicates that the stripped mica is more resistant to corrosion. However, the unmodified mica is less compatible with the resin and unsuitable for long-term corrosion protection. The high open circuit potential of the three composite coatings MC–UIO/EP, MC–UIO@MBT/EP, and PMC–UIO@MBT/EP during the initial phase of immersion can be attributed to the introduction of the amino group of UIO-66, which improves the dispersion of the composite in the epoxy resin and this manifests itself in the coating as a better barrier. The open circuit potential of all composite coatings decreased with increasing immersion time due to the slow penetration of the corrosive media into the interior of the coating, corresponding to a decrease in the shielding effect of the coating. Interestingly, the open circuit potential of MC–UIO@MBT/EP bounced back by 30 days of immersion, a phenomenon attributed to the release of MBT corrosion inhibitor from the coating, which repaired the coating and thus altered the corrosion tendency of MC–UIO@MBT/EP.

**Figure 15:**
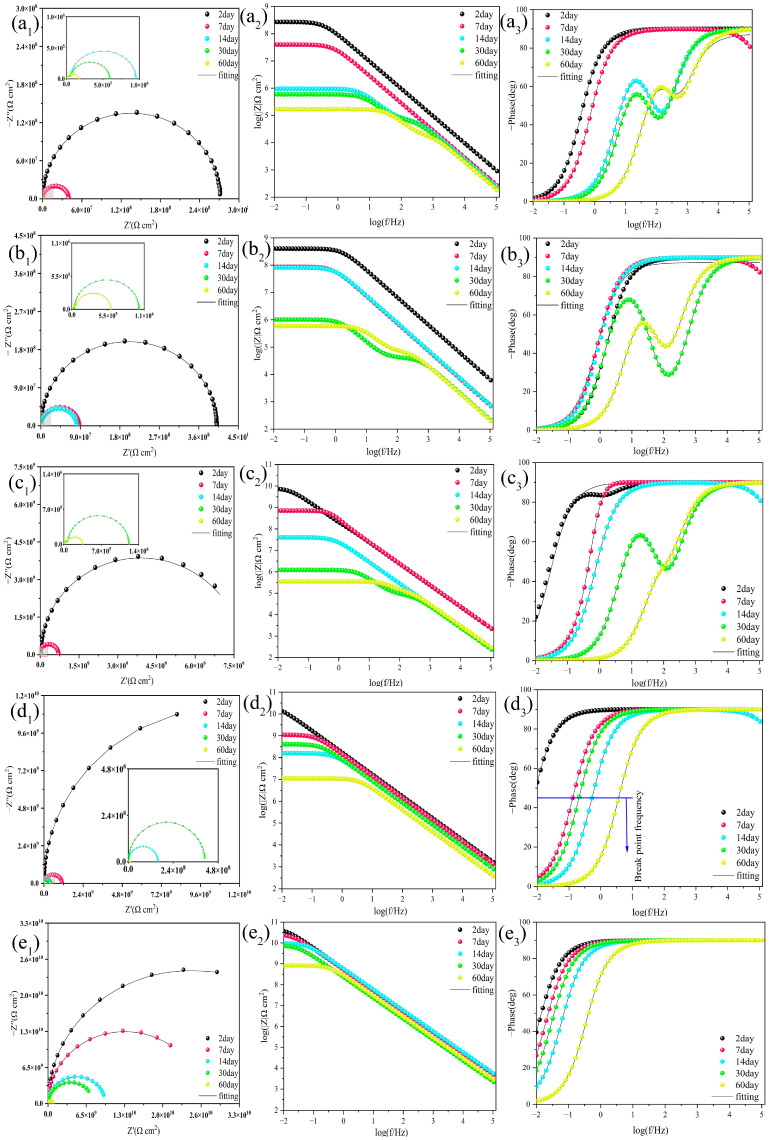
Nyquist, bode, and phase angle plots of (**a_1_**–**a_3_**) NMC/EP, (**b_1_**–**b_3_**) e-MC/EP, (**c_1_**–**c_3_**) MC–UIO/EP, (**d_1_**–**d_3_**) MC–UIO@MBT/EP, and (**e_1_**–**e_3_**) PMC–UIO@MBT/EP at different immersion times in 3.5 wt.% NaCl solution.

Furthermore, the corrosion resistance of the coatings can be judged visually based on the radius of the semicircle in the Nyquist plot, as shown in the Nyquist plots of the different coatings. At the beginning of the immersion period, all the composite coatings showed semicircles with a larger radius on the Nyquist diagram, which indicates that all the composite coatings had a strong corrosion resistance at the beginning of the immersion period. The radii of the semicircles of all the composite coatings in the Nyquist plot show a decreasing trend with increasing immersion time. For the composite coatings NMC/EP (Figure 15(a1)), e-MC/EP (Figure 15(b1)), and MC–UIO/EP (Figure 15(c1)), two capacitive semicircles appear at 14, 30, and 30 days of immersion, respectively, indicating that the corrosive medium has penetrated the interface between the coating and the metal and the coating has lost its corrosion resistance. In contrast, MC–UIO@MBT/EP (Figure 15(d1)) and PMC–UIO@MBT/EP (Figure 15(e1)) remained with only one impedance semicircle in the Nyquist plot from the beginning of the immersion to 60 days. The impedance circle radius of PMC–UIO@MBT/EP is larger than that of MC–UIO@MBT/EP, which is attributed to the modification of the composite by PEI. On the one hand, the introduction of more amines improves the compatibility of the composite with the resin. On the other hand, the PEI acts as a pitting inhibitor, thus improving the corrosion resistance of the coating. Bode diagrams for all composite coatings tested by EIS are shown in Figure 15. Similar conclusions to the Nyquist plots can be drawn in the Bode impedance plots. Natural mica (NMC) and exfoliated mica (e-MC) are corrosion-resistant for a short period after immersion, due to the barrier effect formed by the laminate in the resin. After a period of immersion, however, their low-frequency impedance modulus (|Z| = 0.01 Hz) decreases rapidly, as shown in Figure 15(a2,b2). Figure 15(d2) shows the variation of the low-frequency impedance modulus of MC–UIO@MBT/EP, where the overall trend of impedance at |Z| = 0.01 Hz decreases with increasing immersion time. However, the low-frequency impedance modulus of MC–UIO@MBT/EP increased at 30 days compared to 14 days of immersion, with values increasing from 1.59 × 10^8^ Ω cm^2^ to 4.08 × 10^8^ Ω cm^2^. This is due to the release of the MBT corrosion inhibitor in the composite coating, which improves the corrosion resistance of the MC–UIO@MBT/EP composite coating. The low-frequency impedance modulus (|Z| = 0.01 Hz) of the PMC–UIO@MBT/EP composite coating with the introduction of PEI did not change significantly in the first 30 days of immersion and its value remained at approximately 10^10^ orders of magnitude (as shown in Figure 15(e2)). This also demonstrates the stable corrosion resistance of the PMC–UIO@MBT/EP composite coating. After immersion for up to 60 days, the low-frequency impedance modulus (|Z| = 0.01 Hz) value of 8.30 × 10^8^ Ω is still greater than several other coatings. This result shows that PMC–UIO@MBT/EP has good barrier protection under 3.5 wt.% NaCl immersion. In addition, the protective capacity of several samples can be assessed in the Bode phase angle diagram. Figure 15(a3–e3) shows the Bode phase angle plots for NMC/EP, e-MC/EP, MC–UIO/EP, MC–UIO@MBT/EP, and PMC–UIO@MBT/EP, respectively. There are three types of NMC/EP (Figure 15(a3)), e-MC/EP (Figure 15(b3)), and MC–UIO/EP (Figure 15(c3)). The Bode phase angle diagrams for the composite coatings show that a second time constant appears at 14 and 30 days of immersion, which indicates that the corrosive medium has penetrated the interface between the coating and the metal and that the coating has lost its corrosion resistance. This corresponds to the conclusions obtained from the Nyquist diagram. The breakpoint frequency (f_b_) can be extracted from the position θ = 45° and the value of the breakpoint frequency is related to the corrosion resistance of the coating and the area of the coating to be desorbed. Three samples, NMC/EP, e-MC/EP, and MC–UIO/EP, showed a rapid decrease in breakpoint frequency from the start of the immersion. In addition, the breakpoint frequency of the MC–UIO@MBT/EP samples decreased from 0.53 Hz (14 days) to 0.21 Hz when immersed for up to 30 days. PMC–UIO@MBT/EP samples have a breakpoint frequency of 0.33 Hz after soaking for 60 days, the smallest of all samples. This is explained by the excellent barrier properties and adhesion of this sample’s coating/metal interface [65].

For the EIS results, the fit parameters were fitted and analyzed using Zsimpwin software; the specific values of the fit parameters are shown in Table S1. All fit parameters are shown in the additional material. For the R_c_ values, the samples were permeated by corrosive media and conductive pathways were gradually formed within the coating. As the immersion time increased, the R_c_ values for all samples gradually decreased. At two days of immersion, the R_c_ value for NMC was 2.73 × 10^8^ Ω, which was the lowest value at the beginning of the immersion. In contrast, sample PMC–UIO@MBT/EP showed the smallest trend in R_c_ values and the maximum value of 8.30 × 10^8^ Ω among all samples at the end of immersion. In addition, the same conclusion can be drawn from the charge transfer resistance (R_ct_). This indicates that the synergistic effect of MBT and PEI in the PMC–UIO@MBT/EP composite coating can effectively prevent the rapid penetration of corrosive media and improve the long-term corrosion resistance of the coating.

To assess the effectiveness of the MC–UIO@MBT and PMC–UIO@MBT composite in the coating, the coating was manually scratched and exposed to a 3.5% NaCl solution. The corrosion resistance of the coatings was assessed by the macroscopic morphology of the coating surfaces. Their surface morphology changes are shown in Figure S3. In the e-MC sample, a large amount of yellow–brown corrosion products formed near the scratch area after only 24 h and the scratch area was raised and delaminated around the perimeter. This was caused by the penetration of electrolyte and iron oxidation products into the coating/steel interface. MC–UIO@MBT/EP samples showed a significant improvement owing to the MBT corrosion inhibitor slowing down the onset of corrosion. However, after 48 h of exposure to the MC–UIO@MBT/EP sample, pitting marks could be observed on the surface of the steel sheet. A small amount of blistering was observed in the unscratched area. In contrast, the PMC–UIO@MBT/EP sample showed a smooth coating surface after 48 h exposure and no significant corrosion products were observed in the scratched area. 

#### 2.3.2. EIS Results for Coatings Immersed in an Alkaline 3.5% NaCl Solution

Based on the UV–VIS results, it is known that ligand displacement and hydrolysis reactions in the presence of NaOH solutions lead to structural defects in UiO-66 which result in the more accessible release of the compound. Therefore, the composite coating was immersed in a 3.5% NaCl solution at pH = 11 and tested with EIS.

**Figure 16:**
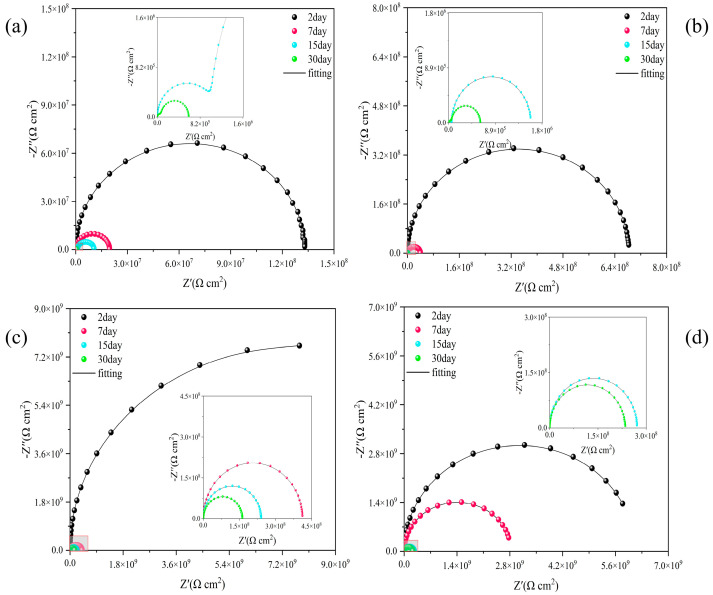
Nyquist plots of (**a**) e-MC/EP, (**b**) MC–UIO/EP, (**c**) MC–UIO@MBT/EP, and (**d**) PMC–UIO@MBT/EP at different immersion times in 3.5 wt.% NaCl solution (pH = 11).

Nyquist plots for each sample are shown in Figure 16 as well as Bode plots in Figure S4. All samples showed a rapid decrease in the radius of the impedance circle under harsh alkalinity conditions, suggesting accelerated coating destruction under harsh conditions. e-MC/EP and MC–UIO/EP samples failed at immersion for up to 15 days. The low-frequency impedance modulus (|Z| = 0.01 Hz) of e-MC/EP decreased rapidly from 1.33 × 10^8^ Ω to 6.02 × 10^5^ Ω while the low-frequency impedance modulus (|Z| = 0.01 Hz) of the PMC–UIO@MBT/EP sample was 2.35 × 10^8^ Ω after 30 days of immersion. This is the maximum value for all samples after 30 days of immersion. This is attributed to the fact that the release of MBT under alkaline conditions in the PMC–UIO@MBT/EP composite coating retards the onset of corrosion. Thus, PMC–UIO@MBT/EP has good corrosion resistance even under alkaline conditions. It can also be shown that the change in pH after the corrosion of a metal surface can promote the release of MBT.

#### 2.3.3. Water Absorption Test of Coatings

**Figure 17:**
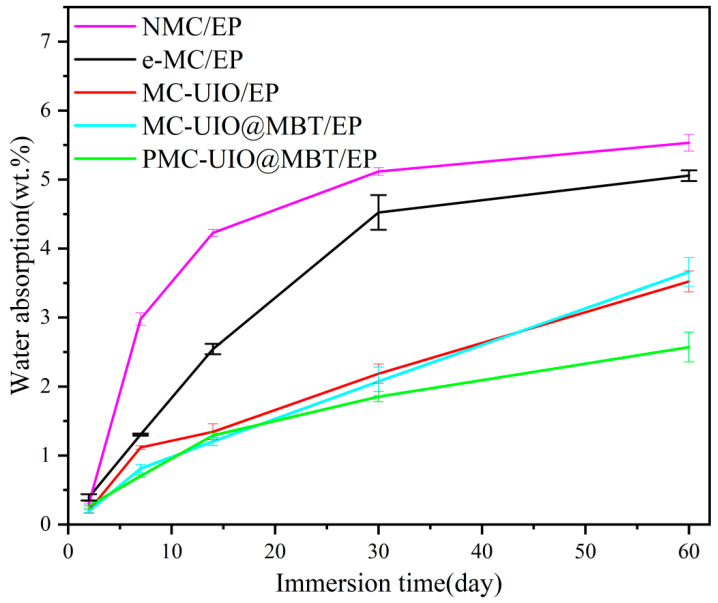
The trend in water absorption of composite coatings after 60 days of immersion.

In practical engineering applications, water absorption is an important indicator to evaluate the barrier performance of a coating. Water absorption is calculated according to the following formula [66]:

(2) $$W_{A}=\frac{M_{t}-M_{0}}{M_{0}}$$

where M_t_ represents the mass of the coated sample at a time (h) and M_0_ represents the mass of the coated sample before the start of soaking. As can be seen in Figure 17, the water absorption of all samples increased with increasing soaking time. NMC/EP shows a rapid increase in water absorption at the beginning of immersion. The aggregation of natural mica and its poor dispersion in epoxy resins results in its inability to seal the micropores formed during high-temperature curing. e-MC/EP samples showed a rapid increase in water absorption in the first 30 days of soaking and reached 5.0% water absorption at 60 days. Combined with the EIS test results, the e-MC/EP samples failed after 60 days of immersion. This is due to many micro-pores caused by the volatilization of organic solvents during the high-temperature curing of the coating and the poor compatibility of the e-MC material in the epoxy resin, which does not act as a barrier to corrosive media in the coating. This is also consistent with the findings of the figure (microporous morphology of the coating). In addition, the water absorption rate of PMC–UIO@MBT/EP samples was 2.57% after soaking for 60 days. This is mainly related to the modification of the composite by the introduction of UIO-66 and polyethyleneimine. Polyethyleneimine and UIO-66 are introduced simultaneously with amino groups, which are beneficial to improve the compatibility of mica in epoxy resins and to play the role of the physical barrier of the laminate in the coating. Therefore, the smaller water absorption of the PMC–UIO@MBT/EP sample can indicate its good corrosion resistance.

## 3. Mechanistic Analysis of Corrosion Resistance

**Figure 18:**
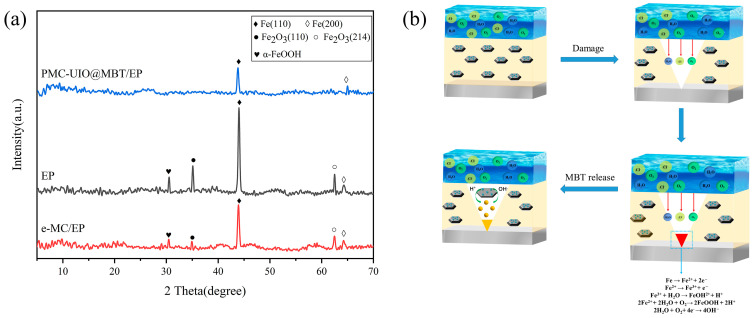
(**a**) XRD of corrosion products. (**b**) The corrosion protection mechanism of PMC–UIO@MBT composite coating.

The main reason for the failure of epoxy coatings on metal substrates is that micro-pores and cracks are formed by the volatilization of organic solvents caused by high temperatures during the curing process. This allows corrosive media to diffuse rapidly through the micro-pores and cracks to the coating/metal interface, leading to corrosion of the metal. The corrosive medium reacts electrochemically on the metal substrate, as shown in Figure 18b. When FeOOH diffuses onto the surface of the coating, this signifies that the metal substrate has corroded. XRD tests were carried out on the composite coating to analyze the corrosion products and the results are shown in Figure 18a. e-MC/EP and the pure epoxy resin have similar positions of characteristic peaks. Both peaks appear at 2θ = 30.5°, which means that FeOOH has appeared and the metal substrate has been corroded. The characteristic peaks at 2θ = 35.1° and 2θ = 62.4° correspond to Fe_2_O_3_(110) and Fe_2_O_3_(214), respectively. The characteristic peaks of e-MC/EP for both Fe_2_O_3_(110) and Fe_2_O_3_(214) are weaker than those of pure epoxy resin. This indicates that the barrier effect of mica in the coating slows down the corrosion rate. All the characteristic peaks in PMC–UIO@MBT/EP are the weakest, which indicates the lightest degree of corrosion on the metal surface. MBT is released in PMC–UIO@MBT/EP due to the H^+^ and OH^−^ generated by the electrochemical reaction of the corrosive medium on the metal substrate and the collapse of the structure of UIO-66 under acidic and alkaline conditions (Fe^3+^ + H_2_O → FeOH^2+^ + H^+^, 2Fe^2+^ + 2H_2_O + O_2_ → 2FeOOH + 2H^+^, 2H_2_O + O_2_+ 4e^−^ → 4OH^−^). In summary, the improved corrosion resistance of the coating can be attributed to the following. (1) The modified composite is better dispersed in the coating, creating a labyrinth effect and extending the path of the corrosive media to the metal substrate. (2) UIO-66 is loaded with an MBT corrosion inhibitor and has a pH-responsive release capability. (3) PEI could resist pitting corrosion.

## 4. Experiment

### 4.1. Material

Nitric acid (HNO_3_), amino-terephthalic acid (C_8_H_7_NO_4_), zirconium tetrachloride (ZrCl₄), acetone (CH₃COCH₃), glacial acetic acid (CH₃COOH), ethyl alcohol (CH₃CH_2_OH), sodium chloride (NaCl), isopropyl alcohol (C₃H₈O), 2-mercaptobenzothiazole (C_14_H_8_N_2_S_4_, MBT), and N, N-dimethylformamide (C_3_H_7_NO, DMF) were purchased from Kelong Chemical Co., Ltd., Chengdu, China. Cetyltrimethylammonium bromide (CTAB) was purchased from Lablead (Beijing, China). Polyethyleneimine (PEI) was purchased from Macklin Group (Coleraine, MN, USA). All the above materials are of analytical purity (AR). The natural mica was purchased from LingShou County, Hebei Province, China from Liming Mineral Products Co. Epoxy emulsion (model number: SM6101, epoxide equivalent: 175–190 g/EP, epoxide value: 0.51–0.54 mol/g). DIW (deionized water) was obtained using a water purifier (UPC-III-40L, Ulupure, Chengdu, China). Comparison of the abbreviations and meanings of all complex products in the synthesis process, as shown in Table 3.

### 4.2. Exfoliation of Natural Mica

The results of related research show that ultrasonic power and time are essential factors in the process of peeling. Moreover, ethanol, as a solvent, is also conducive to the formation of a single-layer mica nanosheet. The degree of solvent is also crucial to the degree of peeling of mica [18]. In the large alcohol solvent of mole, the peeling effect is better [67]. This may be due to the strong solvent of the alcohol and the fact that the intervening spacing of the alcohol molecules is large. There is a hydroxyl group on the surface of the mica. These structures may be the active site conducive to the mica’s surface modification [68].

Pretreatment of natural mica: The mica was calcined at 800 °C and dispersed in a nitric acid solution at a specific concentration. The resulting powder was then dispersed in a saturated NaCl solution at 95 °C and stirred for 3 h. CTAB was then added to intercalate the mica and expand the layer spacing. The product was washed several times by centrifugation with deionized water at medium speed. The intercalated mica was dispersed in an appropriate amount of isopropanol solution and then sonicated at 700 W for 1 h (after continuous sonication for 10 min, the beaker was placed in water to cool and this was repeated 6 times). The sonicated solution was left to stand for 48 h to obtain a supernatant containing a single-layer mica nanosheets or one with a few layers. Finally, the solution was filtered through a medium-speed (3000 rpm) filter and dried under vacuum at 80 °C for 24 h.

### 4.3. Preparation of MC–UIO and Loading of Corrosion Inhibitor

The Mica/UiO-66 composite was synthesized using a one-step synthesis method. The concentration of mica used was 0.1 mg/mL and DMF was used as a solvent. In total, 0.23 g ZrCl_4_, 0.18 g 2-amino terephthalic acid, and 2 mL glacial acetic acid were added to 100 mL of mica dispersed in DMF solution containing 0.1 mg/mL Mica. The mixture was transferred to a Teflon-lined oven and held at 120 °C for 12 h. After centrifugation at 5000 rpm for 20 min, the resulting precipitate was washed three times with DMF and dried. An excess of MBT was added to 100 mL of DMF containing 100 mg of MC–UIO nanocomposite. The mixed solution was stirred at 800 rpm for 24 h while the conical flask was evacuated to make a negative pressure to increase the loading of MBT. The solute was then washed three times with DMF to remove excess MBT from the solution and finally, the product was dried in a vacuum oven at 80 °C for 24 h to obtain the product, named MC–UIO@MBT.

### 4.4. Preparation of PMC@UIO@MBT Composites

**Figure 19:**
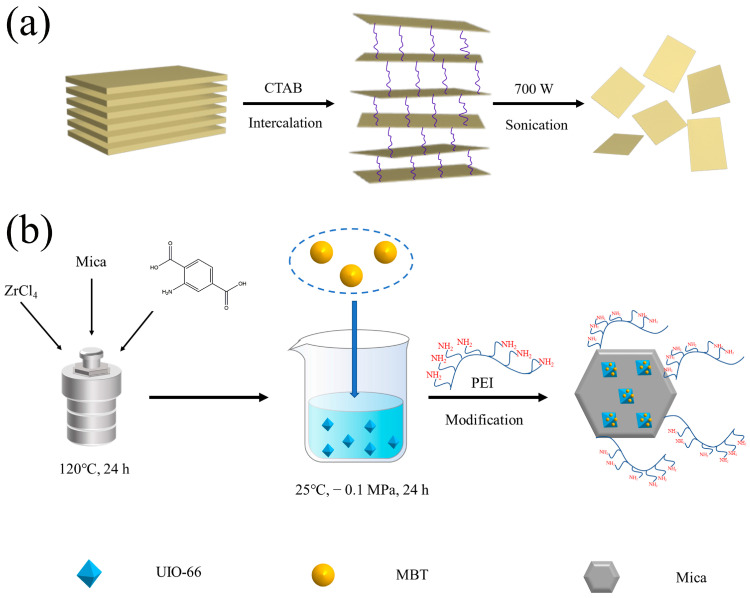
(**a**) Flow chart for exfoliating natural mica; (**b**) Synthesis of PMC–UIO@MBT composite.

The MC–UIO@MBT composites were modified with PEI. In total, 100 mg PEI in an appropriate amount of DIW was subsequently added to the MC–UIO@MBT composites prepared in the previous step. After being homogeneously dispersed by sonication, it was stirred at 80 °C for 8 h. The product was centrifuged and washed several times with DIW and anhydrous ethanol and the resulting precipitate was dried under vacuum at 80 °C. The final product obtained was named PMC–UIO@MBT. The synthesis process of composite materials is shown in Figure 19.

### 4.5. Preparation of Composite Coatings

The coating is prepared as follows: sand the steel sheet to smoothness with 240, 800, and 1000 grit sandpaper and then, ultrasonically clean with acetone and ethanol, respectively. Take 10 g of epoxy resin and 1.5 g of hardener and mix. Then, add 0.05 g of PMC–UIO@MBT composite to the solution and ultrasonicate until the material is completely dispersed in the epoxy resin. The coating was applied to an area of 1 cm^2^ of N80 carbon steel. The coating was left at room temperature for 48 h and then placed in an oven at 80 °C for 12 h.

Using the same preparation method as above, coatings of NMC (natural mica), e-MC (exfoliated natural mica), MC–UIO, MC–UIO@MBT, and PMC/UIO@MBT were prepared. They were named NMC/EP, e-MC/EP MC–UIO/EP, MC–UIO@MBT/EP, and PMC–UIO@MBT/EP, respectively.

### 4.6. Characterization

All the composites were characterized by line diffractometer (XRD), Fourier transforms infrared spectroscopy (FTIR), thermogravimetric analysis (TGA), specific surface area (BET), UV–VIS spectrophotometry (UV–VIS), and scanning electron microscopy (SEM). The performance of the composite coating was tested by electrochemical impedance spectroscopy (EIS), a pull-off test, and a water absorption test. Test methods and equipment models are listed in the Supplementary Materials.

## 5. Conclusions

In this work, ultrasonic exfoliation of natural mica to obtain monolayer or layer-less nano mica flakes was performed and PMC–UIO@MBT composites loaded with corrosion inhibitors were successfully prepared. This is crucial for applying mica as a nano-filler in organic corrosion protection coatings. The main conclusions of this paper are as follows:

(1) The successful preparation of the composites and the successful encapsulation of MBT as a corrosion inhibitor in UIO-66 were demonstrated by characterization tests such as XRD, FTIR, TGA, BET, UV–VIS, and SEM. The release of MBT can be achieved due to the collapse of the crystal structure of UIO-66 under acidic and alkaline conditions. By modifying the material with polyethyleneimine, we discovered that UIO-66 is more densely loaded onto the mica flakes. This facilitates the loading of more corrosion inhibitors, as shown in the SEM results. In addition, adding polyethyleneimine and UIO-66 into the amino group improved the dispersion and compatibility of the composite in the epoxy coating;
(2) In coating performance tests, the corrosion resistance of PMC–UIO@MBT/EP was significantly improved and the coating still had a low-frequency impedance modulus of 8.30 × 108 Ω after 60 days of immersion in a 3.5 wt.% NaCl solution at pH = 7. As well as the change in pH in the cathodic region due to metal corrosion, the MBT is released by the controlled UIO-66 pH response, allowing the coating to recover. Therefore, the highest low-frequency impedance modulus values are also achieved after 30 days of immersion in 3.5 wt.% NaCl solutions at pH = 11;
(3) The relatively small water absorption and the adhesion of PMC–UIO@MBT/EP indicate its good barrier properties and stability. The adhesion of PMC–UIO@MBT coating was increased to 9.01 MPa; water absorption was only 2.57% after 60 days of immersion in a 3.5 wt.% NaCl solution.

## Figures and Tables

**Table 2:** Textural properties of the samples were determined from the adsorption–desorption isotherms of N_2_.

Sample	BET Surface Area (m^2^/g)	Pore Volume (cm^3^/g)	Pore Size (nm)
UIO-66	727.7943	0.6474	3.56
MC–UIO@MBT	452.3264	0.4952	4.38

**Table 3:** Comparison of the abbreviations and meanings of all complex products in the synthesis process.

Abbreviated Name	Implication
MC	Mica
NMC	Natural mica
e-MC	Exfoliated mica
MC–UIO	UIO-66 loaded on e-MC
MC–UIO@MBT	Loading of corrosion inhibitor MBT on MC–UIO
PMC–UIO@MBT	MC–UIO@MBT modified by polyethyleneimine
Abbreviated name/EP	The composites that corresponded were dissolved in epoxy resin to create the coatings

## Data Availability

Not applicable.

## Supplementary Materials

The following supporting information can be downloaded at: https://www.mdpi.com/article/10.3390/molecules28207106/s1, Table S1. Fitting parameters for EIS results measured with composite coatings immersed in 3.5 wt.% NaCl solution for 60 days. Figure S1. Controlled release of MBT from PMC-UIO@MBT under different pH conditions. (a) pH = 3, (b) pH = 7, (c) pH = 11. Figure S2. Photographs of EP, e-MC/EP, MC-UIO/EP, MC-UIO@MBT/EP, MC-UIO@MBT/EP samples for the adhesion pull-off test. Figure S3. Surface appearance of the sample after being manually scratched. Figure S4. Nyquist plots of (a) e-MC/EP, (b) MC-UIO/EP, (c) MC-UIO@MBT/EP and (d) PMC-UIO@MBT/EP at different immersion times in 3.5 wt.% NaCl solution (pH = 11).
