# Supplementary material for: Performance Research of Natural Mica Modified with Zirconium-Based Metal–Organic Frameworks for an Epoxy Resin Anti-Corrosion Coating
Source: Molecules. 2023 Oct 16;28(20):7106. doi: 10.3390/molecules28207106 (PMC10609246; doi:10.3390/molecules28207106)
Supplement: Supplementary file 1 [file molecules-28-07106-s001.zip › molecules-2560828-supplementary.pdf]

Experimental Equipment and Methods

FT-IR (WQF-520) was used to intercept data at wavelengths of 4000 ~ 400 cm^{-1}. x-ray diffraction (XRD, PANalytical, X'Pert PRO MPD) was used with Cu Kα radiation sources scanning from 5° to 70°. Scanning electron microscopy (SEM, JSM-7500F) was used to obtain the surface morphology of the composites for further analysis and to analyse the surface morphology of the coatings. TG (Hitachi TG/DTA7300, Japan, temperature range 25 ~ 800°C, heating rate 20°C/min) is used to analyse the mass changes that occur in composite materials when subjected to heat. The specific area and pore size distribution of the materials were measured by BET (ASAP2460). Sedimentation experiments with H_2O, CH_3CH_2OH, DMF as solvents and a zeta potential analyser were carried out to analyse the change in dispersion of the material after modification by PEI. Characterization of the release behaviour of MBT from PMC-UIO@MBT in NaCl solutions at different pH using a UV-Vis spectrophotometer (UV-Vis, METASD UV-5800).

The corrosion resistance of the coating was tested by electrochemical impedance spectroscopy (EIS) in a conventional three-electrode mode corresponding to a platinum electrode (counter electrode), a reference electrode and a working electrode. The working electrode was coated with 1 cm × 1 cm of N80 carbon steel. The coatings were immersed at room temperature in 3.5% NaCl solutions of different pH. The coatings were subjected to electrochemical impedance spectroscopy for 60 days using a Koster electrochemical workstation (HS310) and equivalent circuits were obtained using Zsimpwin software to analyse the corrosion resistance and mechanism of the coatings. To verify the mechanism of MBT release and retardation in the coating, the coating was manually scratched and the corrosion products on the surface and the extent of coating damage were observed after immersion in a 3.5% sodium chloride solution at room temperature to assess the corrosion resistance of the coating. EIS electrochemical impedance spectra were obtained in the frequency range of 10^5 ~ 10^2 and at a sinusoidal disturbance amplitude of ±10mV. The bond strength of the coating to the metal substrate was demonstrated by pull-off tests using a DeFelsko pull-off instrument (PosiTest- AT), USA.

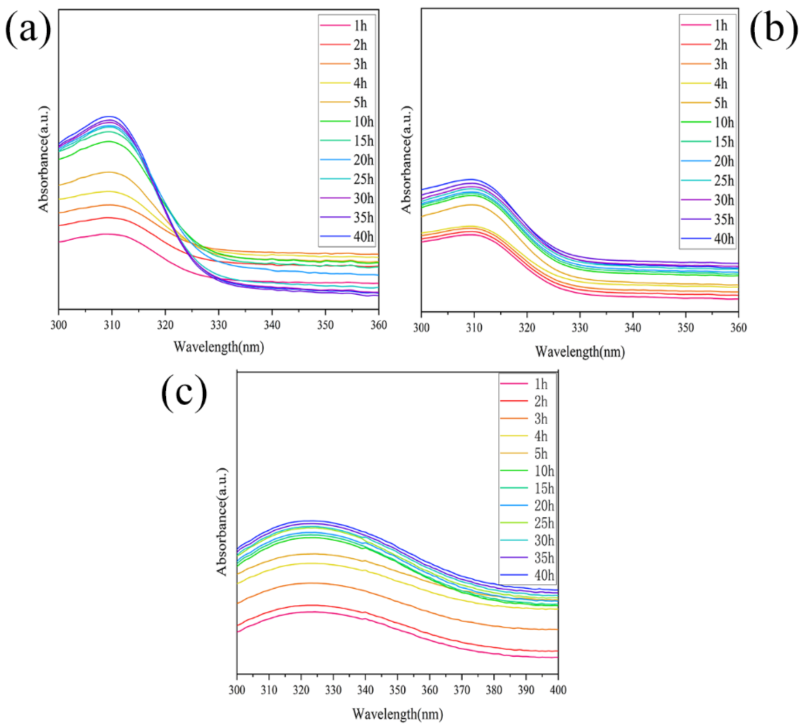

Figure S1. Controlled release of MBT from PMC-UIO@MBT under different pH conditions. (**a**) pH = 3, (**b**) pH = 7, (**c**) pH = 11.

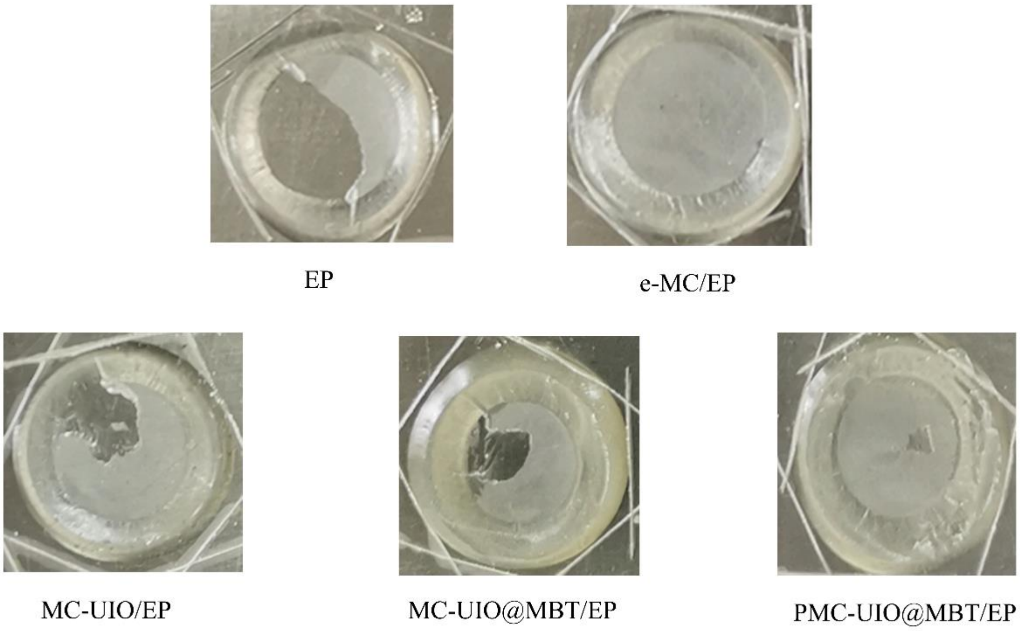

Figure S2. Photographs of EP, e-MC/EP, MC-UIO/EP, MC-UIO@MBT/EP, MC-UIO@MBT/EP samples for the adhesion pull-off test.

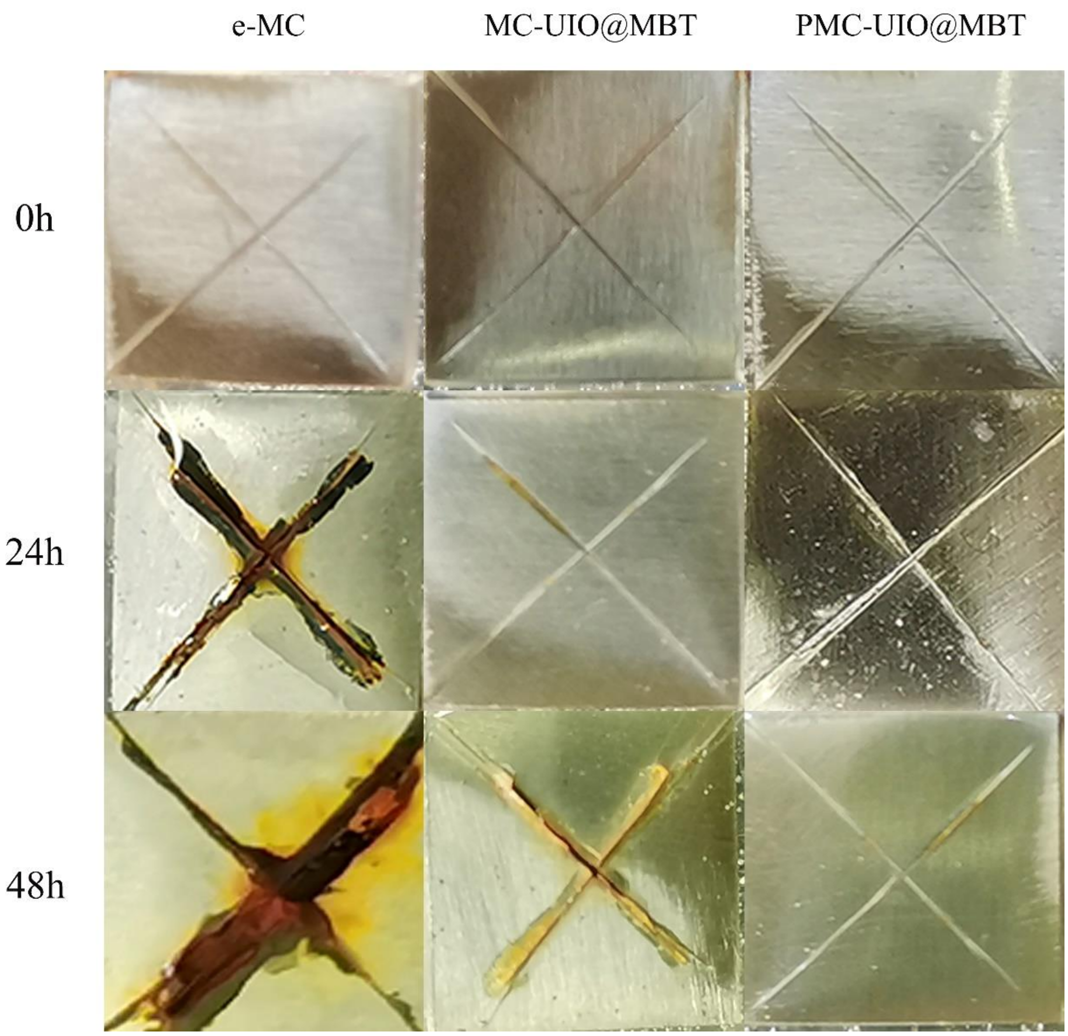

Figure S3. Surface appearance of the sample after being manually scratched.

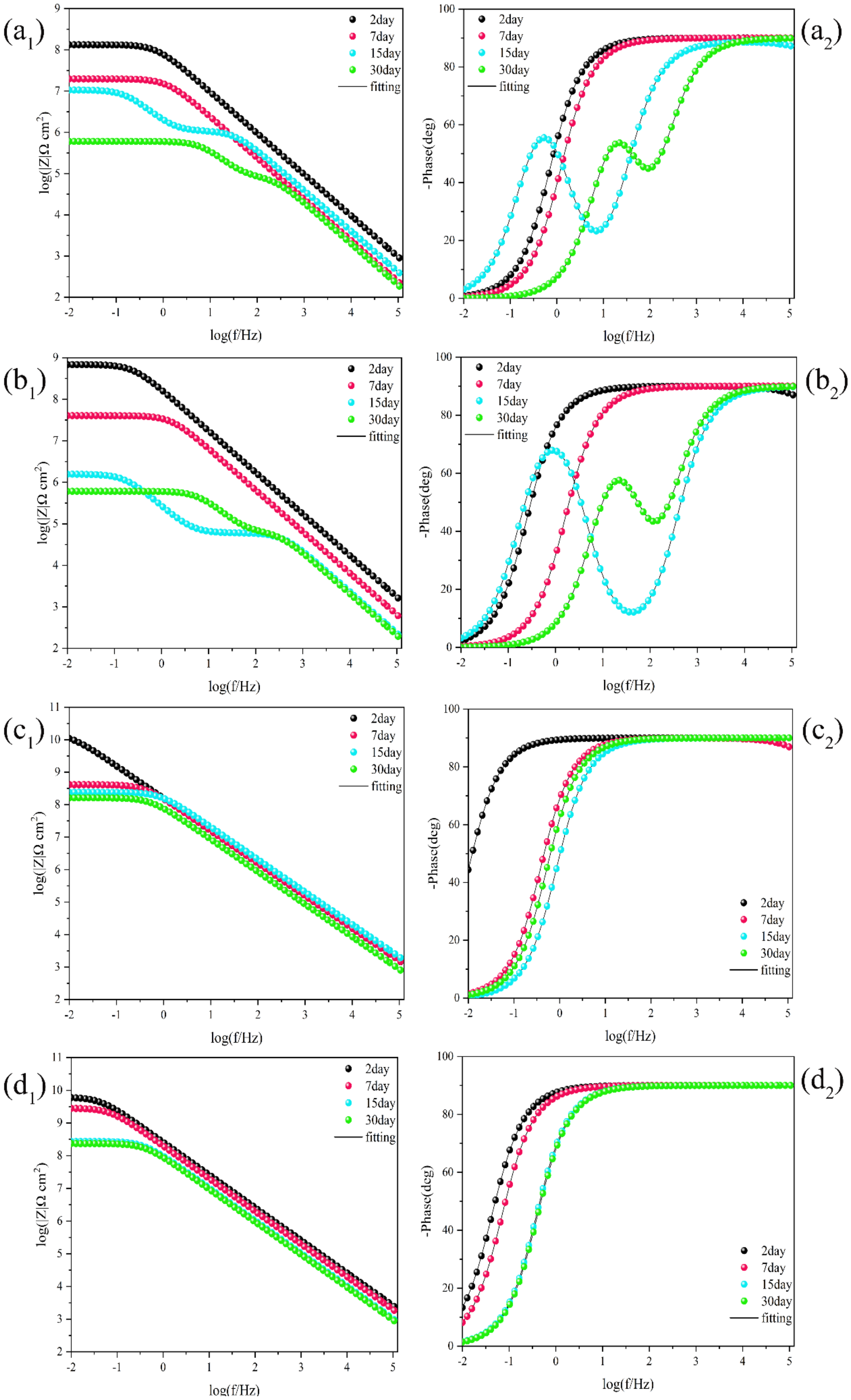

Figure S4. Nyquist plots of (**a**) e-MC/EP, (**b**) MC-UIO/EP, (**c**) MC-UIO@MBT/EP and (**d**) PMC-UIO@MBT/EP at different immersion times in 3.5 wt% NaCl solution (pH = 11).

Table S1. Fitting parameters for EIS results measured with composite coatings immersed in 3.5 wt% NaCl solution for 60 days.

**Sample**	**Immersion Time (Day)**	R_{ct} (Ω cm^2)	CPE_{dl}		R_C (Ω cm^2)	CPE_C	
			Q ($Ω^{-1}$ cm^{-2} s^n)	*n*		*Q* (n$Ω^{-1}$ cm^{-2} s^n)	*n*
NMC/EP	2				2.73e^8	1.73e-9	0.99
	7				4.01e7	6.03e-9	0.98
	15	8.60e5	2.97e-8	0.95	1.03e5	6.66e-9	0.98
	30	4.98e5	3.79e-8	0.87	9.87e4	1.26e-8	0.95
	60	1.41e5	2.46e-8	0.98	2.99e4	1.15e-8	0.97
e-MC/EP	2	-	-	-	4.02e8	2.61e-10	0.99
	7	-	-	-	8.77e7	2.33e-9	0.99
	15	-	-	-	8.13e7	2.95e-9	0.97
	30	9.77e5	9.95e-8	0.94	4.43e4	7.08e-9	0.95
	60	5.10e5	7.53e-7	0.91	9.87e4	1.26e-8	0.95
MC-UIO/EP	2	-	-	-	7.97e9	7.69e-10	0.98
	7	-	-	-	8.26e8	9.33e-10	0.97
	15	-	-	-	3.98e7	5.60e-9	0.99
	30	1.11e7	2.79e-8	0.75	1.08e5	6.31e-9	0.97
	60	1.83e5	1.85e-7	0.94	1.67e5	5.21e-9	0.99
MC-UIO @MBT/E	2	-	-	-	2.25e10	9.36e-10	0.98
	7	-	-	-	1.11e9	1.09e-9	0.99
	15				4.10e8	1.89e-9	0.99

P	30				1.56e8	1.88e-9	0.98
	60				1.11e7	3.91e-9	0.99
PMC-UIO@MBT/EP	2	-	-	-	4.83e10	2.72e-10	0.99
	7	-	-	-	2.61e10	3.08e-10	0.97
	15	-	-	-	9.71e9	2.71e-10	0.99
	30	-	-	-	7.65e9	6.91e-10	0.99
	60	-	-	-	8.30e8	5.48e-10	0.98
